# The role of radiotherapy in adult soft tissues sarcoma of the extremities

**DOI:** 10.1007/s00590-021-02990-6

**Published:** 2021-05-06

**Authors:** Silvia Cammelli, Annalisa Cortesi, Milly Buwenge, Alice Zamagni, Martina Ferioli, Giulia Ghigi, Antonino Romeo, Alessio G. Morganti

**Affiliations:** 1 Radiation Oncology, IRCCS Azienda Ospedaliero-Universitaria di Bologna, Bologna, Italy; 2 Department of Experimental, Diagnostic and Specialty Medicine—DIMES, Alma Mater Studiorum University of Bologna, Bologna, Italy; 3 Radiotherapy Unit, IRCCS Istituto Romagnolo per lo Studio dei Tumori (IRST) “Dino Amadori”, Meldola, Italy

**Keywords:** Soft tissue sarcoma, Radiotherapy, Extremities, Limbs, Review

## Abstract

Local management of adult soft tissue sarcoma of the extremities has evolved over the past decades. Until the 1970s, radical surgery (amputations) was the standard therapeutic procedure resulting in significant physical and psychological morbidity for the patients. In the present era, limb sparing surgery combined with radiotherapy represents the current standard of care for high grade and > 5 cm STSs. This approach guarantees high local control rate and function preservation. The aim of this paper is to summarize the current evidence for RT in STSs of the extremities. Outcomes, technical details (techniques, timing, dose, volumes of treatment) and the emerging role of RT in the management of oligometastatic disease will be analysed. Finally, results of the recent clinical trials testing new scenarios in RT of STSs will be described.

## Introduction

Soft tissue sarcomas (STSs) are a rare and heterogeneous group of tumours originating from connective tissue, representing 1% of all adult and 15% of paediatric malignancies [1]. Adult STSs originate mostly from the extremities (43%), followed by visceral (19%), retroperitoneum (15%), trunk (10%) and head and neck (9%) [1].

More than 50 different histological and molecular subtypes of STS have been described by the World Health Organization (WHO) among which the most common subtypes in adults are undifferentiated pleomorphic sarcoma, liposarcoma and leiomyosarcoma [2]. Prognosis depends on histological subtypes, grading, size, tumour location, presence of distant metastases and other factors [2].

Extremity and trunk STSs show about 60% of disease-specific survival at 10 years; local recurrence rates range from 20 to 30% and 30–50% of cases develop metastases (most frequently in the lungs). The incidence of distant metastases at diagnosis is about 10% and is more likely in patients with large, deep and high-grade lesions.

Radiotherapy (RT) plays an increasing role in the multimodal treatment of soft tissue sarcomas. Technological evolutions like intensity-modulated, image-guided and stereotactic radiotherapy have considerably increased the potential of radiotherapy in the last 20 years, leading to significant improvements in patient management. In fact, they have allowed either dose escalation (with consequent improved efficacy) or reduction in toxicity (with consequent improved functional outcome).

The primary aim of this paper is to summarize the state of the art about the role of RT on extremity STSs of adult. Furthermore, the impact of RT on local control (LC), overall survival (OS), outcomes in oligometastatic presentation, and future direction about RT application in clinical practice will be analysed.

RT in bone sarcomas [3], sarcomas typical of paediatric and adolescent age (like rhabdomyosarcoma and Ewing sarcoma), sarcoma originating from visceral organs and head and neck, as well as sarcoma-like lesion (like dermatofibrosarcoma protuberans and desmoid fibromatosis) will not be described in this review.

## Role of radiotherapy on local tumour control

Surgery is the mainstay of treatment for resectable STSs. Until the 1970s, the standard surgical procedure was amputations.

In 1982, the prospective randomized trial conducted by Rosemberg et al. [4] compared patients to receive either amputation or limb sparing surgery plus adjuvant RT. Twenty-seven patients were randomized to receive limb-sparing resection and RT, 16 patients received amputation (randomization was 2:1). The authors found no statistically significant differences in OS (*p* = 0.99) and LC (*p* = 0.06) rates: 5-year OS and LC for amputation and limb-sparing surgery plus RT were 88% versus 83% and 100% versus 85%, respectively.

Thereafter, several studies have investigated the role of RT on tumour LC, suggesting a positive effect [5, 6]. According to these evidences, the addition of RT to conservative surgery is currently recommended in international guidelines [1], 2]. The combined modality is associated with a local recurrence rates lower than 15% [5–7].

Two randomized studies compared conservative surgery alone versus surgery combined with adjuvant RT [5, 6]. Yang et al. reported results from 141 patients affected by STSs, 99 high-grade (G2-G3), and 50 low grade (G1). Patients were randomized to receive adjuvant external beam RT (EBRT) or not, after conservative surgery. With a median follow-up of 9.6 years, a statistically significant improvement in LC was recorded for high grade sarcomas treated with adjuvant EBRT vs surgery alone: 10-year LC were 100% vs. 78%, respectively (*p* = 0.003). A trend was recorded in low grade sarcomas in favour of patients treated with EBRT vs surgery alone: 10-year LC were 95% vs. 68%, respectively (*p* = 0.067). These data were confirmed in a recent update:[7]: after a median follow-up of 17.9 years, local recurrence rate was 25% following limb sparing surgery alone compared to 1.4% in those treated with adjuvant EBRT (*p* = 0.0001).

Pisters et al. randomized 164 patients to receive conservative surgery + adjuvant brachytherapy (BRT) vs surgery alone. With a median follow-up of 76 months, patients with high-grade sarcomas treated with BRT presented statistically higher 5-year LC rates than patients with low grade lesions: 89% versus 66%, respectively (*p* = 0.0025). BRT had no impact on LC in patients with low-grade lesions (*p* = 0.49).

Data from the largest retrospective study conducted by Jebsen et al. [8] (1093 adult patients) indicate a benefit from adjuvant RT regardless of the state of margins. The benefit was more pronounced in deep-seated and high-grade tumours. In this group of patients, the risk of local recurrence without RT was more than three times greater than that with RT. The 5-year local control rate was 28% without RT and 62% after RT for intralesional margin, 74% versus 81% for marginal margin and 87% vs. 93% after wide margin.

Major literature data about local control in extremities STSs are described in Table 1.

**Table 1:** Summary of outcome from major studies

References	Study design	Setting	No. of patients	Median F/U (years)	5 year LC (%					5 year DFS (%)	5 year OS (%)
Yang et al. [5]	Randomized	LSS alone	71	17.9	78 (10 year)	High grade		68 (10 year)	Low grade	Not reported	74 (10 year)
		LSS + post-op EBRT	70		100 (10 year)			95 (10 year)			75 (10 year)
O'Sullivan et al. [17]	Randomized	Pre-op EBRT	94	3.3			*p* = 0.791			Not reported	*p* = 0.791
		Post-op EBRT	96								
Jebsen et al. [8]	Retrospective	LSS alone	598	5	28 (Intralesional margins)		74 (Marginal margins)	87 (Marginal margins)		Not reported	Not reported
		LSS + EBRT (pre or post)	381		62 (Intralesional margins)		81 (Marginal margins)	93 (marginal margins)			
Pisters et al. [6]	Prospective randomized	LSS alone	164	6.3			69			81	Not reported
		LSS + BRT					82			84	
Sampath et al. [19]	Retrospective	Pre-op EBRT	293	5.3			93			Not reported	65
		Post-op EBRT	528				87				60
Wang et al. [27]	Prospective phase II	Pre-op EBRT + LSS	79	3.6			94 (2 year)			61.5 (2 year)	80.6 (2 year)
Koshy et al. [10]	Retrospective	LSS alone	3.689	Not reported			Not reported			Not reported	73 (3 year)
		LSS + EBRT (pre or post)	3.271								63 (3 year)
Alektiar et al. [21]	Retrospective	LSS + EBRT (pre or post)	41	3			84			Not reported	64

EBRT: external beam radiotherapy, LSS: limb sparing surgery

Based on these evidence, international guidelines recommend addition of RT to limb sparing surgery in patients with high grade, large (> 5 cm), deep lesion. For low grade lesions, RT is usually reserved for patients with positive margins or local recurrence without prior RT. In selected cases, it can be useful in a preoperative setting even in Grade 1 lesion to help the surgeon to have adequate margins.

## Role of radiotherapy on overall survival

From the available literature, RT impact on survival is unclear. The two prospective randomized trials by Yang et al. [5] and Pisters et al. [6] did not demonstrate an impact of adjuvant RT on OS.

Two subsequent retrospective studies from the surveillance, epidemiology, and end results (SEER) database [9, 10] have shown different conclusions.

Kachare et al. [9] analysed 2606 high grade extremities sarcoma patients divided in two cohorts, created using propensity score matching between irradiated and non-irradiated groups. In the final analysis, RT was associated with survival advantage in both univariate and multivariate analysis.

Kohsy et al. [10] included 6960 patients in their analysis: in high-grade tumours, 3-year OS in patients who received RT and those who did not was 73% versus 63%, respectively (*p* < 0.001), supporting the benefit of RT on survival.

Although these data are encouraging and are in favour of a wide use of RT, they need to be interpreted with caution. The retrospective study designs and the fact that SEER registry does not provide data that could have an impact on outcomes, such as the use of chemotherapy or information on RT technique, might have influenced the results.

Major literature data about overall survival in extremities STSs are described in Table 1.

## Role of combined chemo-radiation therapy

There is no consensus on the current role of combined radiation and chemotherapy for adult extremities STSs. Study results are conflicting about this topic, and consequently, it cannot be considered the standard treatment.

A meta-analysis published in 2008 found a statistically significant benefit in terms of both relapse-free survival and OS in the adjuvant setting [11]. A randomized trial published afterwards [12] did not confirm these results showing no benefit from adjuvant EBRT with doxorubicin and ifosfamide in both relapse free survival and OS.

The literature data about neoadjuvant setting are controversial as well. Only one randomized trial comparing pre-operative chemotherapy and surgery with or without radiotherapy versus surgery with or without RT alone was done and did not demonstrate any advantage from adding systemic treatment [13]. Another study found a significant benefit in overall survival for neoadjuvant chemotherapy plus hyperthermia [14].

As result of these conflicting data, differences in clinical practice regarding use of adjuvant and/or neoadjuvant chemotherapy worldwide are relevant. It can be proposed as an option to high risk cases (high grade, deep, > 5 cm tumour) with sensitive histological types. The decision should be shared in a multidisciplinary setting taking into account the potential increased toxicity of the combined treatment. About this topic, literature data [15] reported high rate of toxicity (5% grade 5, 83% grade 4) in patients received three cycles of neoadjuvant chemotherapy (modified mesna, doxorubicin, ifosfamide, and dacarbazine [MAID]), interdigitated preoperative radiation therapy (RT; 44 Gy administered in split courses), and three cycles of postoperative CT (modified MAID). In the study published by Palassini et al. [16], the combined radio-chemotherapy (epirubicin plus ifosfamide) treatment was found to be feasible, safe and with a limited increase in toxicities compared to patients receiving preoperative CHT alone.

## Techniques

### External beam radiation therapy (EBRT)

The standard of radiation treatment after limb sparing surgery was originally the adjuvant setting [4, 5]. However, the indications for preoperative RT are increasing due to the various advantages compared to postoperative RT [17, 18]. Several recently published studies have evaluated the benefits and risks of neoadjuvant and adjuvant RT treatment [17, 18].

O’Sullivan et al. [17] from the Canadian Sarcoma Group, conducted a phase III randomized study to compare both approaches [17]. One hundred and ninety patients were enrolled, 94 in pre-operative arm and 96 in post-operative. The primary end point was the rate of wound complications within 120 days of surgery. After a median follow-up of 3.3 years, they did not observe any statistically significant difference in LC and progression free survival rates between the two groups. However, important differences with regard to side effects were recorded: preoperative RT was associated with greater incidence of acute wound complications compared to post-operative RT, 35% versus 17%, respectively (*p* = 0.01). On the other hand, late side effects like fibrosis, edema and joint stiffness were more common in patients receiving post-operative RT. The revised data at prolonged follow-up (5 years) confirmed these results [18].

Sampath et al. published the largest retrospective comparison between pre-operative and post-operative RT [19] conducted on 821 patients. The primary aim was to assess the impact of RT sequencing with surgery on OS and LC. With a median follow-up of 5.2 years, better outcomes on local and distant control were found in the pre-operative setting. Five-year local failure free survival was 93% and 87% in the pre-operative and in the post-operative group, respectively (*p* < 0.05). Five-year overall survival was 65% and 60% in the pre-operative and post-operative group, respectively (*p* < 0.01). Despite the retrospective nature of this study, the authors suggested the hypothesis that pre-op RT may provide patients with the opportunity to improve long-term survival.

Based on the available literature data, it is possible to affirm that both treatments, pre-operative and post-operative, lead to similar results in terms of LC and OS and that the main differences are in terms of side effects.

As already discussed, the most frequents pre-operative EBRT related side effect are wound complications (frequency 19.5–35%) [6]. The internal side of the thigh, tumour size > 10 cm, the tumour proximity to skin surface are risks factor for the development of this unwanted side effect. [7, 8]. Major literature data about EBRT related acute and late toxicity in extremities STSs are described in Table 2**.**

**Table 2:** Summary of side effects from major studies

References	Study design	Setting	No. of patients	Median F/U (years)	Wound complications (%)	Late toxicity grade ≥ 2 (%)		
O'Sullivan et al. [17]	Randomized	Pre-op EBRT	94	3.3	35	Not reported		
		Post-op EBRT	96		17			
Davis [18]	Randomized	Pre-op EBRT	73	Not reported	Not reported	31.5 Fibrosis	15.1 Edema	17.8 Joint stiffness
		Post-op EBRT	56			48.2	23.2	23.2
Wang et al. [22]	Prospective phase II	Pre-op EBRT + LSS	79	3.6	36.6	10.5		
Beane et al. [7]	Randomized	LSS alone	71	17.9	20	4 Bone fracture	12 Edema	
		LSS + post-opEBRT	70		27	10	25	
Alektiar et al. [21]	Retrospective	LSS + EBRT (pre or post	41	3	19.5	4.8 Bone fracture	12 Edema	17.1 Joint stiffness

EBRT: external beam radiotherapy; LSS: limb sparing surgery

**Fig. 1:**
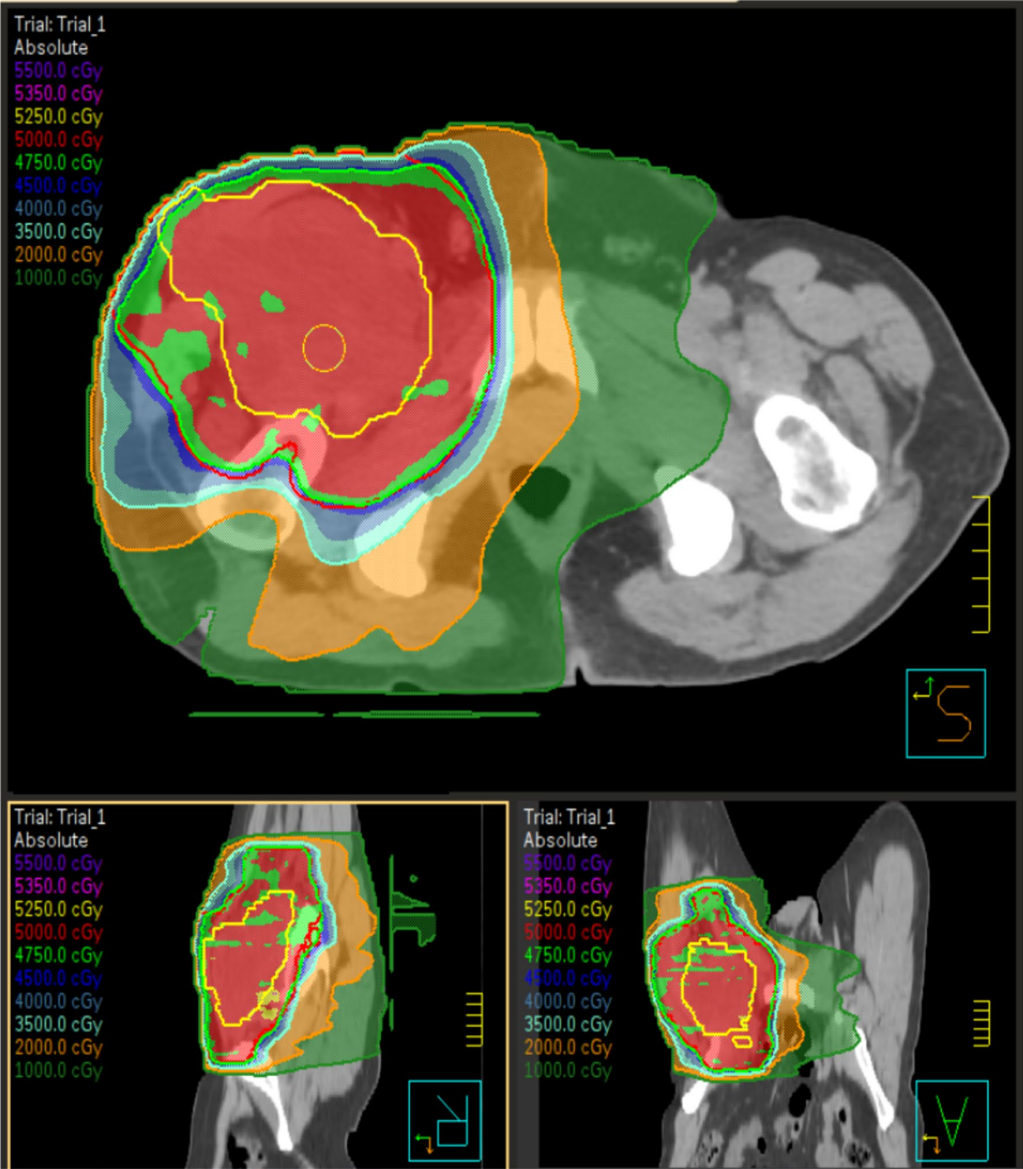
example of isodose distribution for a patient with soft tissue sarcoma of the thigh treated with volumetric modulated arc therapy (VMAT)

In clinical practice, the indication for each treatment must be evaluated in multidisciplinary committee, considering the optimal sequence between surgery and RT, taking into account various factors for each patient (tumour size, anatomical position of the disease, histological subtype, patient's comorbidity, risk factors). For both treatment settings, the evolution of RT techniques has led to an improvement in the planning of RT and a consequent reduction in short- and long-term toxicity. From commonly used 3D-CRT techniques, in the recent era, there has been an evolution towards intensity-modulated radiation therapy (IMRT) and volumetric modulated arc therapy (VMAT), techniques that involve the use of variable intensity multiple beams: they allows a more conformal dose distribution to the target volumes (Fig. 1), a reduction in locoregional failure [20], an excellent local control in patients with high risk features and a greater sparing of neighbouring healthy tissues [21].

#### Preoperative EBRT

The main advantages of preoperative EBRT are that the total radiation dose is lower than in the post-operative setting (50 Gy vs. 60–66 Gy) [17] and treatment volumes are frequently smaller with consequently lower late toxicity and improved long-term functional outcomes [22]. Furthermore, the definition of the target volumes is easy, as the disease is clearly visible in magnetic resonance imaging (MRI) [23]. Moreover, there is an increase in radiobiological efficacy due to the better oxygenation and vascularization of the tumour. Finally, from a theoretical point of view, surgical resection could become easier due to the possible shrinkage and cellular modification of the pseudocapsule diseases).

The patients who can benefit most from neoadjuvant EBRT are those who present deep disease, greater than 5 cm, high grade and where surgery is complex due to the proximity of STS to neurovascular bundle or bone [23].

Preoperative RT also has clear advantages both for surgery and for local control in patients affected by myxoid liposarcoma, in consideration of the high rate of shrinkage/size reduction that the tumour can achieve [24].

Main disadvantage of neoadjuvant EBRT is high rate of surgical wound complications. The randomized trial by O’Sullivan et al. (3) reported 35% of wound complications in the preoperative group versus 17% in the postoperative group. Another possible disadvantage of preoperative RT is potential risk for patients unresponsive to radiation treatment to progress during RT, making them unable to receive definitive surgery.

Surgery should be performed 4–6 weeks after the end of RT, to reduce the risk of wound complications and limit acute reactions [23]. However, it is not advisable to wait too long due to the risk of development of late fibrosis that could hinder surgery.

Currently, the standard dose for neoadjuvant EBRT is 50 Gy in 25 fractions. The overall RT period is 5 weeks.

In case of positive surgical margins, the possibility of performing an adjuvant boost using EBRT or BRT (14–20 Gy is described in the international guidelines [1]. However, the results of two retrospective trials [25, 26] showed that postoperative RT boost did not clearly provide any advantage in preventing local relapse in patients with positive surgical margins. Furthermore, the advantage of adding postoperative RT boost has not yet been evaluated in randomized clinical trial. The topic is highly debatable and risk of local failure vs. potential toxicity should be very carefully evaluated individually for each patient.

**Fig. 2:**
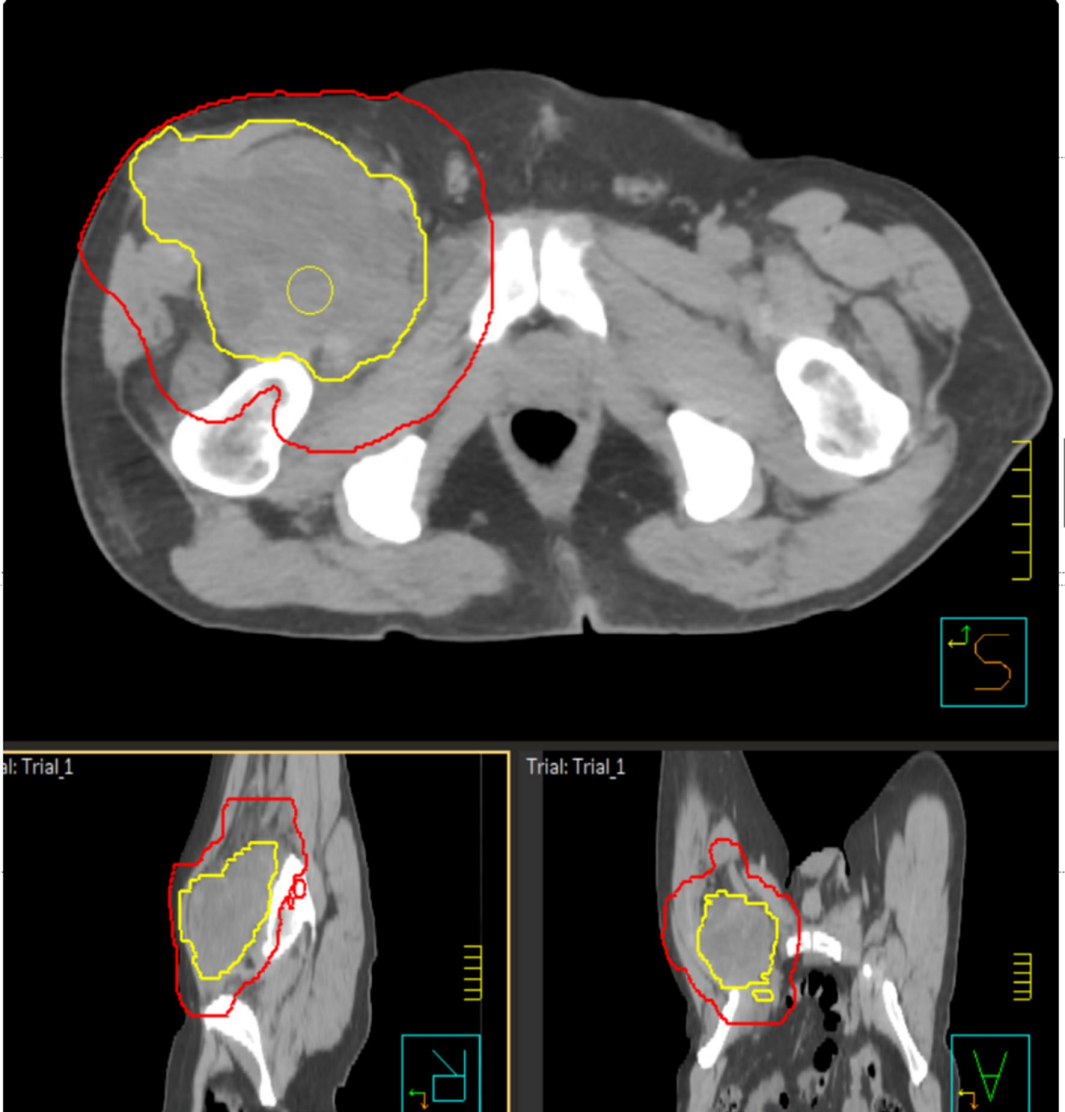
Example of target delineation for a patient with soft tissue sarcoma of the thigh. Yellow line represents gross tumour volume (GTV). Red line represents planning target volume (PTV)

A consensus on the definition of gross target volume (GTV) and clinical target volume (CTV) was obtained by the Radiation Therapy Oncology Group (RTOG) [27] and by Haas and his European, American and Canadian colleagues [23]. GTV is defined on T1 contrast-weighted MRI images; fusion of CT and MRI images is recommended for treatment planning. CTV is defined as GTV + microscopic clinical extension: currently, it is obtained with an expansion of 3 to 4 cm longitudinal margins and 1.5 cm radial margins to GTV. CTV extension can be limited to the anatomical compartment limits. Planning target volume (PTV) corresponds to an isotropic expansion of 5–10 mm of the CTV (Fig. 2); it varies according to the immobilization systems, the techniques and the image-guided RT used in each center [28].

#### Postoperative EBRT

Postoperative RT allows a definitive pathologic assessment. Moreover, it is associated with a lower rate of scar and postoperative wound healing complications. It is especially indicated in patients with significant comorbidities, at risk of wound complications.

On the other hand have to be considered several disadvantages of post-operative RT: larger target volumes need to be irradiated than neoadjuvant RT and higher total dose are requested. Thus, postoperative RT is associated with a higher rates of late toxicity than preoperative RT. Side effects such as fibrosis, joint weakness, bone fracture, oedema are frequently permanent and can consequently lead to a reduction in patient's quality of life [17].

Currently, the standard dose for adjuvant EBRT is 50 Gy with standard fractionation to a larger volume encompassing the surgical bed with appropriately safe margins, followed by a boost to the tumour bed of 10–20 Gy if surgical margins are not adequate, resulting in a total dose of 60–70 Gy.

CTV must include tumour bed, all surgically manipulated tissues, all visible metal clips, the entire surgical scar and extent of the operative field and drain sites, with a 3.5 to 4 cm of longitudinal and 1.5 cm of radial margins, with the exception of osseous planes or fascia that act as natural barriers [29]. It is recommended to keep a drainage area radio protected to reduce distal oedema and late severe complications [23]. The boost target volume should be created according to the original tumour extension. To define it accurately, it is necessary to provide a pre-operative CT or MRI data set. In post-operative setting, positioning of metal surgical clips during excision by surgeon is crucial to permit radiation oncologist to define the target volume in the proper manner [23] For the delineation of PTV, an isotropic expansion of the CTV of 5–10 mm is given.

### Brachytherapy

After a recent systematic literature review and clinical experiences, American guidelines on the use of BRT in STS have been drawn up [30] and previous American Brachytherapy Society guideline [31] has been updated. Guidelines reported use of BRT as RT-boost in cases at high risk of recurrence.

Andrews et al. reported a study [32] involving 130 patients, 25 treated with BRT with a mean dose of 16 Gy (range 10–20 Gy) + EBRT with a dose of 50 Gy (range 40–70 Gy), while 61 patients were treated with exclusive EBRT with a median dose of 59 Gy (range, 50–74). Five-year OS, MFS and LC for patients treated with BT + EBRT vs EBRT alone were 82% vs. 72% (*p* = 0.93), 90% vs. 78% (*p* = 0.15) and 90% vs 83%, respectively (*p* = 0.25). In the univariate analysis, improved 5-year LC was found for high-grade tumours when treated with BRT + EBRT instead of EBRT alone, 100% vs 74%, respectively (*p* = 0.09). These data suggested that the combination of BRT to EBRT may offer a better LC than EBRT alone for large deep-seated high-grade STS.

In the early 2000s, with the introduction of technological advancement in EBRT, BRT started to be replaced by EBRT techniques such as IMRT. Between 1995 and 2006, a large retrospective study comparing adjuvant BRT to IMRT was conducted at Memorial Sloan-Kettering Cancer Center by Alektiar et al. [33]. One hundred and thirty-four adult patients affected by high grade extremities STS were enrolled: 71 received BRT, 63 received EBRT using IMRT technique, both in adjuvant setting. LC was significantly better in the IMRT group compared to the BRT one.

A large series on adjuvant EBRT plus BRT boost in STS was recently performed [34]: 107 patients affected by high grade primary or recurrent STS underwent adjuvant BRT 20 Gy + EBRT 46 Gy ± chemotherapy. Five-year LC and OS rates were 80.9% and 87.4%, respectively.

BRT as a monotherapy can be considered in low-risk diseases, in case of small high-grade STS with negative margins or for re-irradiation. In fact, BRT should also be considered for patients with STS relapse who have previously undergone limb-sparing surgery and EBRT, and where complications increase after re-treatment of previously irradiated tissues [35].

### Intraoperative radiation therapy (IORT)

IORT consists of the delivery of a high dose of radiation in a single fraction during surgical procedure to the tumour bed in order to obtain an enhanced biological effect reducing toxicity, being able to move radiosensitive structures at risk out of the radiation field.

Updated recommendations on the use of intraoperative RT (IORT) have recently been published [36], in the light of an updated systematic review of literature.

Experts recommend IORT as a possible alternative to EBRT boost after pre-operative RT with R1 or R2 resection. Furthermore, it can be used as part of the post-operative RT treatment. Post-operative RT, as already mentioned, is usually divided in two part: a dose of 50 Gy prescribed to an extended PTV, followed by a RT dose of 10–20 Gy to a limited PTV (based on surgical margin status). The latter ‘‘boost” phase can be replaced by a preceded IORT boost. In both cases, the main advantage is the smaller irradiated volume with consequent potential lower side effects.

Several studies in literature have shown how the combination of limb sparing surgery, IORT and EBRT proved very high 5-year LC rates, 82–97% [37], 38, furthermore associated with high limb preservation rates and good functional outcomes [37, 38]. The good functional results are probably due to the fact that radiosensitive structures or organs at risk (like major nerves or skin) can be spared from the field of irradiation. Furthermore, since there are no problems of intra- and inter-fraction movements, margins can be reduced to a minimum, allowing to deliver high dose to a much smaller irradiated volume compared to an EBRT boost [39].

Sometimes IORT is used in case of recurrent extremity STS. Tinkle et al. [38] reported a retrospective analysis of 26 patients with locally recurrent STS who underwent IORT (median dose 15 Gy, range 10–18 Gy) after salvage limb-sparing resection, reporting 58%, 81% and 50% 5-year LC, amputation-free, and OS, respectively, with acceptable morbidity.

### Hadron therapy

Hadron therapy is a form of EBRT using beams of energetic carbon ions, protons, or other heavier positive ions. The most common type of particle therapy is proton therapy. The chief advantage of proton therapy is that the RT dose is delivered over a narrow range of depth, which results in minimal entry, exit, or scattered radiation dose to healthy nearby tissues. Indeed, particle beams exhibit a Bragg peak [40] in energy loss through the body, delivering their maximum radiation dose at or near the tumour and minimizing damage to surrounding normal tissues [41].

Hadron therapy does not represent the standard of RT in extremity STS because modern technologies of conventional EBRT are satisfactory. However, some patients affected by proximal thigh or trunk sarcomas of big dimension could benefit from proton therapy for better healthy tissues sparing.

Proton therapy is up to date the standard of care for the skull base, spinal and paraspinal sarcomas. Moreover, it is often indicated in case of bone sarcomas, pediatric diseases, retroperitoneal or large volume sarcomas, second tumours or for re-irradiation in case of relapses.

Guttmann et al. [42] conducted a prospective study on 23 patients with locally recurrent or new primary STS in previously irradiated fields who underwent proton re-irradiation. Treatment was generally well tolerated with low high-grade toxicity rates. Therefore, they concluded that proton re-irradiation for STS may be considered a possible and safe treatment option.

Gary et al. from Loma Linda University are running a phase II single arm trial (NCT01819831) investigating the role of preoperative proton therapy (total dose 50 Gy, 25 daily fractions) followed by limb sparing surgery. The primary end point of the study is late radiation toxicities > grade 2 at 2 years.

## Role of radiotherapy in oligometastatic disease

Unfortunately, natural evolution of STSs is characterized by a high risk of distant metastasis (DM). Approximately 50% of STS patients develop DM [43] even in case of optimal primary treatment. The risk is higher for those patients with large tumour and/or with high grade histology. The most common site of DM is the lung (70–80%) followed by bone, liver and brain.

The approach to metastatic STS depends on several factor, such as number and site of metastases, histological subtype of primary tumour, interval between primary tumour diagnosis and development of DM, performance status and presence of comorbidities. Even if chemotherapy remains the standard of care for metastatic sarcoma patients, lack of durable responses and limited effect from systemic therapy highlights the importance of local treatment in the management of metastatic STS.

Increasing literature evidence suggests, in selected subgroup of patients affected by oligometastatic disease, a benefit of locally ablative treatments (often in combination with systemic agents) resulting in longer life expectancy and better quality of life [44]. An additional advantage in the use of local ablative therapy could be its role in delaying initiation or change of systemic therapy. The largest retrospective study about this topic was published by Falk et al. [44], reporting improved OS for patients who received local ablative treatment (surgery, RT, radiofrequency ablation) of lung, liver or other metastases site compared to patients who do not received any local treatment (*p* = 0.0001).

Historically, surgery of metastatic site has been considered the treatment of choice. Instead, RT has been mainly used for palliation and to reduce risk of bone fracture and cord compression. Recent technological advances in RT offer the opportunity to change the role of this modality in the strategic approach to oligometastatic STS. The selection of patients who can benefit is complex, and it depends on several factors and should always require a multidisciplinary approach.

One technical possibility for DM-directed RT is stereotactic body RT (SBRT). It is characterized by highly focused radiation to small volume in a single or few large dose fractions. This hypofractionated regimen is more biologically effective than conventionally fractionated RT. Furthermore, it can help to overcame radiation resistance, typical of radioresistant tumour such as STS.

The study with the largest number of patients was published by Lindsay and colleagues [45]. They treated 117 metastatic soft tissue and bone sarcoma lung nodules in 44 patients. With a median follow-up of 14 months, 2-year OS and pulmonary LC were 82% and 95%, respectively; only six out of 177 pulmonary nodules showed progression. Twenty-five percent of patients reported radiation-associated complications graded as ≤ 3 except one patient who developed an oesophageal stricture.

Other literature series also reveal favourable outcomes and confirm low toxicity profile supporting SBRT as a valid alternative to surgery, not only for lung (Table 3) but also for other sites such as bone [46] and brain [47]

**Table 3:** Published series about SBRT for sarcoma lung metastases

Authors	Year	No. of patients	No. of lesions	Total dose/ No. of fractions	Median F/U (months)	2 years L.C (%)	2 years O.S (%)	Toxicity Gr ≥ 3 (%)
Dhakal et al. (preferred)[55]	2012	14	74	50 Gy/5 fr	11	88	69	None
Frakulli et al.[56]^†^	2015	24	68	30–60 Gy/3–8 fr	17	86	66	None
Navarria et al. [57]	2015	28	51	48 Gy/4 fr (preferred)	21	96	56	None
Soyfer et al. [58]	2017	22	53	60 Gy/3 fr	95	100*	50*	n.r
Lindsay et al.[45]^†^	2018	44	117	50 Gy/10 fr (preferred)	14	95^‡^	82	2
Baumann et al.[59]^†^	2020	56	44	50 Gy/4–5 fr	16	90	46	None

*5 year rate

^†^These studies included patients with both soft tissue and bone sarcomas

^‡^Local pulmonary control rate

## Future directions

The current local approach for extremities STSs, based on limb sparing surgery and RT, has permitted to reach satisfactory results in terms of local recurrence rate (lower than 15%) [5, 6, 8]. Furthermore, advancement in RT field leads to two main objectives: reducing treatment associated acute and late side effects and increasing the efficacy of RT. To reach the first objective, the introduction of IMRT and image-guided radiation therapy has allowed to better conform irradiated volume and to use smaller safety margins resulting in reduced side effect.

The recent phase II RTOG 0630 trial [22] demonstrated reduced grade 2 late toxicity rates (11%) compared to 37% reported in the historical randomized NCIC Trial. Eighty-six patients were treated in a preoperative setting (50 Gy in 25 fractions) with reduced margin around the macroscopic lesion compared to that reported in International guidelines [23].

Another field of research, aimed at saving resources, involves shortening radiation schedule using hypofractionated RT regimen. Hypofractionation represents a kind of RT fractionation in which the total dose is delivered in fewer fractions with an increased fraction dose. This treatment may lead to additional biological effects compared to conventionally fractionated RT. The main advantages of hypofractionation are the decreased overall treatment time which is more convenient for both patients and physicians, the increased compliance and the best cost-effectiveness ratio of the treatment. Furthermore, such an approach may provide an additional radiobiological benefit when treating radioresistant tumours such as sarcomas.

Results published by Kosela-Paterczyk et al. [48] are encouraging and suggest a possible use of this approach in clinical practice. They treated 272 patients with pre-operative RT for five consecutive days in 5 Gy per fraction, immediately followed by surgery. LC was comparable to previously published studies (81%); early toxicity was similar to the Canadian study by O’Sullivan (32.4% vs. 35%), but only 7% of patients required surgery for treatment of the complications (vs. 16% in the Canadian study); late toxicity was 16% (vs. 32% in the Canadian study [17]).

The high interest on this topic is confirmed by several trials currently underway in this area. At least six are now registered at www.clinicaltrials.gov and are currently accruing patients. One of them, the running trial NCT03989596 conducted by Maria Sklodowska-Curie Institute—Oncology Center, is investigating hypofractionated pre-operative RT schedule (10 fractions × 3.25 Gy) combined hyperthermia in marginally resectable STSs followed by a RT boost 4 × 4 Gy within one week in case of unresectability.

Another field of research is studying effects of intratumoural injections of radio-enhancing substances in order to increase the radiobiological effects of radiation with minimized systemic side effects. Bonvalot et al. [49] recently published encouraging results for intratumoural injection of nanoparticles of hafnium oxide (NBTXR3) [49]. They conducted a phase II-III randomized trial comparing standard pre-operative RT (50 Gy) versus standard pre-operative RT preceded by NBTXR3 injection into the primary tumour site. They reported pathological complete response in 14 out of 87 patients (16%) in the NBTXR3 group and in 7 out of 89 patients (8%) who underwent RT alone, (*p* = 0.044), and a higher rate of R0 resections (77% vs. 64%, *p* = 0.042) in the NBTXR3 group. They did not record significant differences in severe side effects. Based on these preliminary data, although they have to be confirmed by long-term analysis and further trials, this could be considered as a possible new treatment option.

Another aspect often debated among sarcoma scientific community is the possibility to treat different histological subtypes by specific treatment approaches, also including tailored RT treatment schedule. The rationale is based on the knowledge that the big group of these rare solid tumours of STS includes many histological subtypes with many differences in biological and clinical behaviour. For example, myxoid liposarcoma has been reported in several studies as characterized by higher radiation sensitivity. It shows greater reduction in size and greater pathological response to RT compared to other histological subtypes [50].

On the basis of this finding, the recent international prospective phase II DOREMY trial [51] was conducted in order to assess whether a dose reduction in preoperative RT for myxoid liposarcoma would result in comparable oncological outcome to the present standard of care with less morbidity. Seventy-nine patients who received a preoperative RT dose of 36 Gy in once-daily 2 Gy fractions were enrolled. Extensive pathological treatment response was observed in 91% of patients; with a median follow-up of 25 months, rate of grade 2 or higher toxicity was 14%, lower compared to the 37% of the historical Canadian study [17]. Based on these evidence, authors propose the use of 36 Gy (2 Gy/fraction) as an alternative approach of pre-operative RT for myxoid liposarcoma. This represents the first attempt to histology-stratified RT approach for STS.

Finally, another recent field of investigation is about combined approach of RT with new systemic therapy like target agents or checkpoint inhibitors. Encouraging preliminary data regarding efficacy of combined treatment of RT with, Sorafenib [52], Pazopanib [53] and Sunitinib [54] have been recently published, although some of them are accompanied by unexpected high toxicity. Lewin and colleagues [54] studied a combination of RT (28 fractions,1.8 Gy/die) with sunitinib: 44% of patients presented grade 3 hepatotoxicity rate. However, tumour necrosis was higher in patients treated with combined treatment compared to the group with RT alone (75% vs. 40%) even if higher local failure rate was apparent in patients receiving sunitinib. Several clinical trials are currently on going about this topic and results, to validate role of combined treatment with new agent, are awaited. 

## Conclusions

Conservative surgery combined with RT represents the standard of care for local treatment of high grade STSs of limbs. RT can be administered both pre- or post-operatively. Post-operative modality increases late side effects like fibrosis, oedema and joint stiffness. Pre-operative setting is associated with higher rate of wound complications, but literature evidence suggests that advanced techniques can decrease these complications.

Current guidelines increasingly support the use of neoadjuvant RT, although the optimal timing should be evaluated individually and should involve a multidisciplinary team. Furthermore, there is an increasing and promising RT role in the oligometastatic setting. New approaches like the possible use of shorter fractionation regimen, use of radiosensitizers, tailored RT according to histological subtype are possible fields for future investigations. Finally, due to the rarity of STSs, its management is recommended based on decision making process within multidisciplinary teams at referral Centers.
